# Real-Time Three-Dimensional Echocardiographic Assessment of Severity of Mitral Regurgitation Using Proximal Isovelocity Surface Area and Vena Contracta Area Method. Lessons We Learned and Clinical Implications

**DOI:** 10.1007/s12410-015-9356-7

**Published:** 2015-08-25

**Authors:** Thomas Buck, Björn Plicht

**Affiliations:** Medical Clinic III, Department of Cardiology, Klinikum Westfalen, Am Knappschaftskrankenhaus 1, 44309 Dortmund, Germany

**Keywords:** Color Doppler real-time three-dimensional echocardiography, Vena contracta area, Proximal isovelocity surface area, Mitral regurgitation

## Abstract

Mitral regurgitation (MR) is considered the most common valve disease with a prevalence of 2–3 % of significant regurgitation (moderate to severe and severe) in the general population. Accurate assessment of the severity of regurgitation was demonstrated to be of significant importance for patient management and prognosis and consequently has been widely recognized in recent guidelines. However, evaluation of severity of valvular regurgitation can be potentially difficult with the largest challenges presenting in cases of mitral regurgitation. Real-time three-dimensional echocardiography (RT3DE) by the use of color Doppler has the potential to overcome the limitations of conventional flow quantification using 2D color Doppler methods. Recent studies validated the application of color Doppler RT3DE for the assessment of flow based on vena contracta area (VCA) and proximal isovelocity surface area (PISA). Particularly, the assessment of VCA by color Doppler RT3DE led to a change of paradigm by understanding the VCA as being strongly asymmetric in the majority of patients and etiologies. In this review, we provide a discussion of the current state of clinical evaluation, limitations, and future perspectives of the two methods and their presentation in recent literature and guidelines.

## Introduction

Valvular insufficiencies are among the most frequent heart diseases [1, 2], and mitral regurgitation (MR) is considered the most common valve disease with a prevalence of 2–3 % of significant regurgitation (moderate to severe and severe) in the general population [1]. Accurate assessment of the severity of regurgitation was demonstrated to be of significant importance for patient management and prognosis and consequently has been widely recognized in recent guidelines [3–6]. However, assessment of severity of valvular regurgitation can be potentially difficult with the largest challenges presenting in cases of mitral regurgitation. Among a variety of different criteria and parameters used for the evaluation of MR severity, flow quantification has become the cornerstone. However, as important accurate flow quantification is, as difficult is it. Over the past decades, multiple invasive and non-invasive methods have been explored and applied to measure mitral regurgitant flow. Since spectral Doppler and later color Doppler were introduced, echocardiography has more and more become the clinical standard method for MR flow analysis. However, accuracy of flow quantification is limited because of complex spatial and dynamic pattern of flow across the mitral valve. Three-dimensional echocardiography which has grown up to a clinically accepted technique has been demonstrated to provide important information for flow quantification and, thus, is promising to overcome the major limitations of 2D-based methods [7]. Recent studies validated the application of color Doppler real-time three-dimensional echocardiography (RT3DE) for the assessment of flow based on vena contracta area (VCA) and proximal isovelocity surface area (PISA). Particularly, the assessment of VCA by color Doppler RT3DE led to a change of paradigm by understanding the VCA as being strongly asymmetric in the majority of patients and etiologies. As a consequence, this has also led to appropriate recognition and changes of recommendations in current guidelines. In this review, we provide a discussion of the current state of clinical evaluation, limitations, and future perspectives of the two methods and their presentation in recent literature and guidelines. This review is also an update of the 2014 review on real-time three-dimensional echocardiographic flow quantification in valvular heart disease [7] but with a focus on mitral regurgitation.

## Basic Principle of Flow Quantification in Valvular Heart Disease

The basic principle of flow quantification exists of the accurate measurement of flow velocity and the cross-sectional area of flow, the two multiplied providing flow rate, and flow rate integrated over time providing flow volume. However, while measurement of velocity and cross-sectional area of laminar flow through a geometric tube is relatively straight forward, the two measurements become very challenging in the situation of flow resulting from a blood volume moved from one chamber of the heart to another by passing through a diseased heart valve like in mitral regurgitation [8]. Blood flow passing through a diseased heart valve is characterized by three important features: (I) an asymmetric cross-sectional area of flow, (II) an irregular-shaped field of flow or flow jet distal to the heart valve in a heart chamber filled with blood, and (III) a complex dynamic pattern of the flow as a result of a combination of driving forces and variable anatomic pattern of the heart valve during the cardiac cycle [7]. From this, it becomes obvious how challenging measurement of flow in the heart by means of the ultrasound technique is. In the following sections, the basic principles and clinical applications of the two clinically most recommended and used methods, the vena contracta area method and the proximal isovelocity surface area method, are described with a special focus on the impact of the application of current RT3DE and their application for the assessment of severity of mitral regurgitation.

## Basic Principle and Clinical Application of Vena Contracta Area Method Using Color Doppler RT3DE in Mitral Regurgitation

**Fig. 1:**
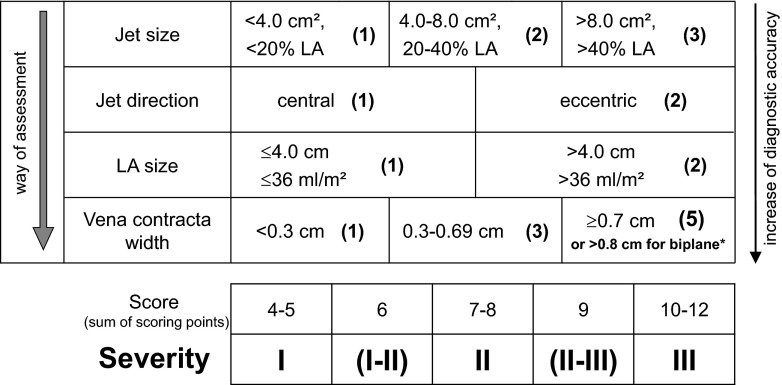
Scoring system for estimation of the severity of mitral regurgitation based on current international recommendations (modified from [21]; Copyright Urban and Vogel). Individual scores for each of the parameters are indicated in *parenthesis*. Summing up the four scores of the four parameters results in total score (sum of scoring points) where each total score is matched with a grade of severity (*bottom*). Note that in patients with asymmetric vena contracta shape a biplane measurement in a 2- and 4-chamber view with a biplane vena contracta width of >0.8 cm has been recently recommended to define severe MR [4, 6]. *LA* left atrium

**Fig. 2:**
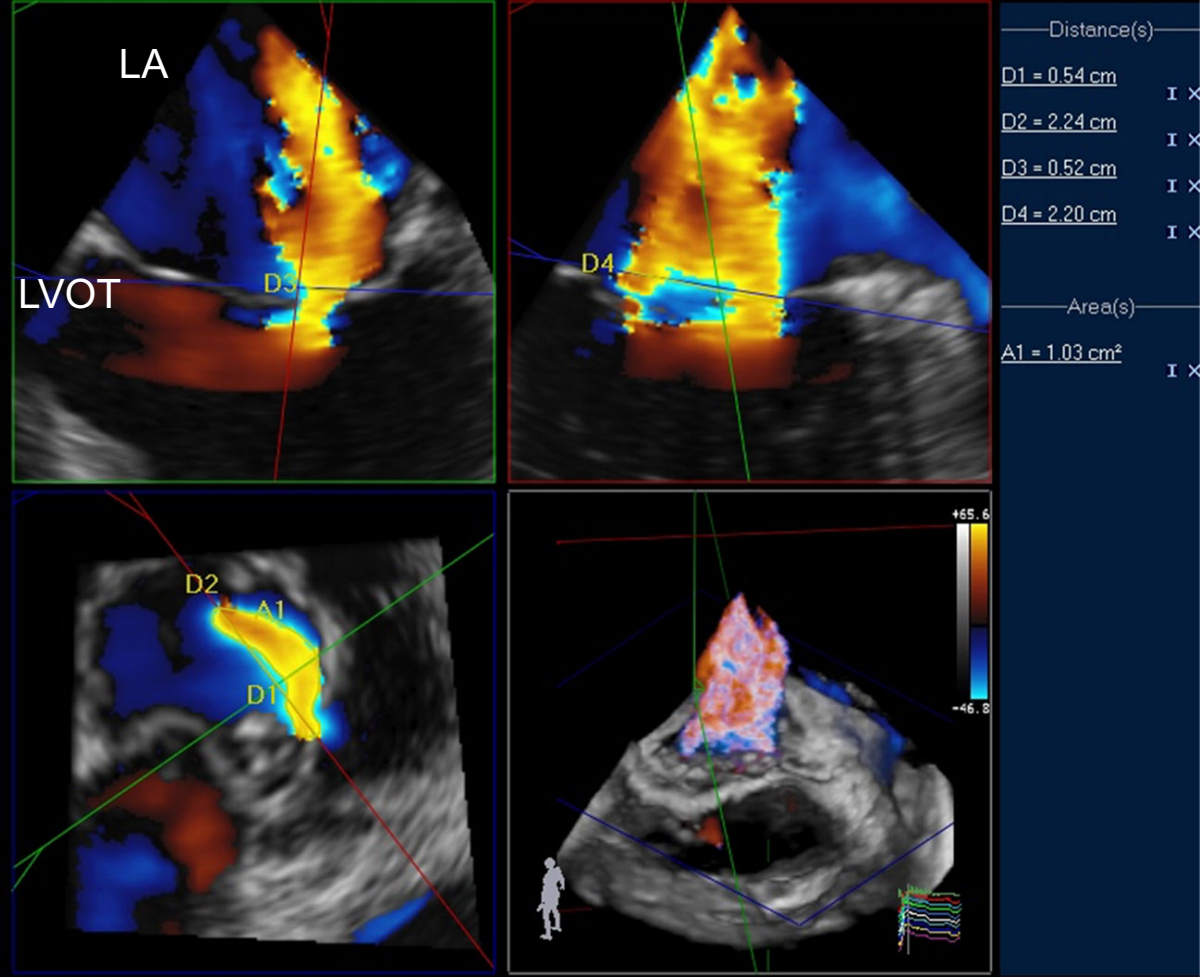
RT3D color Doppler TEE dataset in a patient with severe functional MR. Display of 3D analysis software (Qlab 9.0, Philips Medical Systems) showing a 3D view to the mitral valve and the MR jet from an LA perspective (*bottom right*) and three reconstructed image planes in orthogonal orientation to the MR jet: long-axis LVOT view (*top left*), 2-chamber view (*top right*), short-axis view showing the asymmetric VCA (1.03 cm^2^ by direct planimetry, short-axis diameter (*D1*) = 0.54 cm, long-axis diameter (*D2*) = 2.24 cm) along the commissure line (*bottom left*). *LA* left atrium, *LVOT* left ventricular outflow tract

**Fig. 3:**
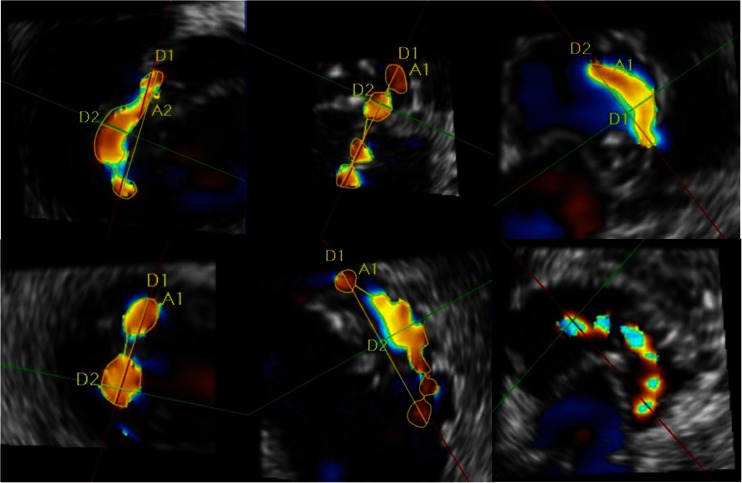
Illustration of the interindividual variability of shape, size, and number of VCAs represented in RT3D color Doppler TEE en face views to the VCA. This figure also illustrates the measurement of multiple VCAs using 3D analysis software (Qlab 9.0, Philips Medical Systems)

As a practical approach to effective regurgitant orifice area (EROA), which corresponds hemodynamically to the cross-sectional area of the vena contracta (VC) as the narrowest portion of the proximal regurgitant jet [8–10], the VC width (VCW) of a color Doppler jet has become an accepted quantitative parameter for estimating MR severity [4–6, 11–14]. However, this simplified assumption of the VCW only holds when the EROA is nearly circular, and recent studies have indicated that the EROA is non-circular in most patients [14–17, 18••], particularly when the VCW at the same time appears narrow in the 4-chamber view and broad in the 2-chamber view as in most cases of functional MR due to incomplete mitral leaflet closure [19]. Nonetheless, the VCW is still an accepted and recommended parameter for the estimation of severity of mitral regurgitation [4–6, 11, 20] and part of an integrative approach of different semiquantitative and quantitative 2D and color Doppler parameters for the estimation of MR severity [3–6]. This integrative approach can be practically applied in clinical routine for grading MR severity by using a standardized scoring system (Fig. 1) [21]. In 2004, color Doppler RT3DE was demonstrated to provide a three-dimensional volume dataset that contains the full anatomic information of the color Doppler flow jet in comparison to two-dimensional color Doppler imaging presenting the flow jet only incompletely in a cross-sectional image plane [16]. Using special analysis software, a color Doppler RT3DE dataset can be cropped to provide a direct en face view to the VCA of a flow jet or image planes in any orientation (“anyplane mode”) can be reconstructed from the dataset providing best presentation of the VCA and VCW (Fig. 2) [22]. Alternatively, the 3D dataset can also be tomographically sliced for accurate identification of the level of the VCA [23]. Khanna et al. initially demonstrated color Doppler RT3DE as a feasible method to provide direct visualization and planimetry of the VCA of a regurgitant jet [16]. Kahlert et al. first proved that RT3DE overcomes the limitations of 2D measurements of VCW by direct assessment of the size and shape of the VCA and demonstrated the differences in VCA asymmetry among different etiologies of MR [18••]. In the majority of patients with functional MR, RT3DE showed typical elongation of the VCA along the semilunar-shaped line of coaptation particularly in cases of incomplete mitral leaflet closure due to leaflet tethering. The variability of shape, size, and number of VCAs in a spectrum of patients with both functional and organic MR is demonstrated in Fig. 3 [23]. Several recent studies compared 3D VCA measurements with other methods particularly for the quantification of mitral regurgitation and demonstrated an increasing superior accuracy of 3D measurements compared to 2D measurements the more asymmetric the VCA was. Subsequent studies provided further validation of RT3DE assessment of the asymmetric VCA by comparison against independent methods [24, 25] and the proof of superiority of 3D VCA measurements compared to 2D VCA measurement in both central and eccentric jets [26] as well as in multiple jets [27]. An overview of recent clinical studies in which 3D VCA measurement has been compared with other methods of MR quantification is provided in Table 1. In all studies, the correlation between direct 3D measurement of VCA and 2D methods was good, but 2D methods, particularly 2D VCW and hemispherical PISA, systematically underestimated the true EROA the more elliptic or asymmetric it was [18••, 24–30].

**Table 1:** Overview of clinical studies validating 3D vena contracta area measurement against 2D methods and independent methods

Study	No. of patients	Scan method	Etiology	Comparison method	Correlation/agreement (mean diff. ± SD)	Inter-/intraobserver variability
Khanna et al.(2004) [16]	44	TTE	Not reported	Ventriculographic grading	*r* = 0.88; limits of agreement not reported	*r*² = 0.99/ *r*² = 0.97
Iwakura et al. (2006) [28]	109	TTE	FMR 63 %	EROA by 2D PISA; EROA by 2D QD	*r* = 0.93 with 2D PISA; 0.07 ± 0.1 cm² *r* = 0.91 with 2D QD; 0.05 ± 0.1 cm²	8.6 %/9.0 %
Kahlert et al. (2008) [18••]	57	TTE	FMR 36 %	EROA by 2D and 3D PISA	*r* = 0.96 with HE PISA; −0.09 ± 0.14 cm² *r* = 0.93 with HSPISA; −0.2 ± 0.20 cm²	0.04 cm²/–
Little et al. (2008) [24]	61	TTE	FMR 44 %	EROA by 2D QD	*r* = 0.85; limits of agreement not reported	0.03/0.05 cm²
Yosefy et al. (2009) [26]	49	TTE	FMR 58 %	EROA by 2D QD	*r*² = 0.86; 0.04 ± 0.06 cm²	0.03/0.02 cm²
Marsan et al. (2009) [29]	64	TTE	FMR 100 %	RVol by CMR	*r* = 0.94 (bias: −0.08 ml, limits of agreement 7.6 ml/−7.7 ml)	0.06/0.04 cm²
Shanks et al. (2010) [25]	30	TEE	FMR 53 %	RVol by CMR	Not reported; 63.2±41.3 ml (3DE) vs. 65.1±42.7 ml (CMR)	0.01/0.01 cm^2^
Zeng et al. (2011) [30]	83	TTE	FMR 47 %	Integrated 2DE methods	*r* = 0.88; limits of agreement not reported	0.03/0.04 cm²
Hyodo et al. (2012) [27]	60	TEE	FMR 100 %	EROA from 3D left ventricular volume and thermodilution data	*r* = 0.90; −0.05 ± 0.06 cm²	0.06/0.05 cm²

*HS* hemispheric, *HE* hemielliptic, *FMR* functional mitral regurgitation, *QD* quantitative Doppler, *RVol* regurgitant volume, *CMR* cardiac magnetic resonance, *EROA* effective regurgitant orifice area, *2DE* two-dimensional echocardiography, *3DE* three-dimensional echocardiography, *PISA* proximal isovelocity surface area

As a consequence, RT3DE assessment of the VCA has significantly changed the understanding of the anatomy of the VCA, which indeed led to a change of paradigm in the assessment of mitral regurgitation severity [31]. Based on the VCA measurements obtained by RT3DE, Kahlert et al. proposed a larger cut-off value of 0.6 cm^2^ for the VCA compared to 0.4 cm^2^ for 2D-derived EROA by the PISA method and accordingly 0.8 cm for mean VCW (mean of 4- and 2-chamber views) instead of 0.7 cm for 4-chamber-based VCW for severe MR for all etiologies, including functional MR [18••], thus overcoming the practical limitation of having a proposed cut-off value of 0.2 cm^2^ for severe functional MR [32] and 0.4 cm^2^ for severe organic MR [5]. This concept of the asymmetric VCA with new cut-off values particularly the 0.8 cm for mean VCW to define severe MR was recently adopted by the guidelines of the European Society of Cardiology [4, 6, 20]. However, as the direct measurement of the VCA using RT3DE is currently still performed manually and only few studies exist investigating VCA cut-off values of MR severity, no cut-off values for VCA have been recommended yet. Zeng et al. proposed a lower cut-off value of the VCA of 0.41 cm^2^ for differentiation of moderate from severe MR that can be applied in all etiologies and orifice shapes [30]. As a potential explanation for the difference between the two cut-off values, Kahlert et al. [18••] derived their 3D cut-off value of 0.6 cm^2^ by correcting the prior 2D-based cut-off value for the underestimation of the true asymmetric VCA by 2D methods, whereas Zeng et al. [30] derived their 3D VCA cut-off value of 0.41 cm^2^ from MR grading based on an integration of conventional 2D methods including 2D PISA, 2D VCW, and 2D jet area. As these two first studies come to significantly different cut-off values for VCA to define severe MR, further studies ideally evaluating the clinical and prognostic value of VCA cut-off values in longitudinal observations are vitally needed.

Accurate measurement of VCA by RT3DE also provides increased accuracy of the estimation of MR flow volume as calculated from the VCA by RT3DE times the velocity-time integral of regurgitant flow by continuous wave Doppler [25, 29]. Marsan et al. validated this as a practical approach and found excellent correlation with regurgitant volume measured by velocity-encoded cardiac magnetic resonance (CMR) (*r* = 0.94) without significant difference between the two techniques (mean difference = −0.08 ml/beat) [29]. Compared to this, 2D echocardiographic assessment of MR volume using VCW in the 4-chamber view significantly underestimated regurgitant volume (*p* = 0.006) as compared with CMR. As for the 3D VCA measurement itself, further validation of 3D VCA-based MR volume calculation is needed.

While RT3DE has overcome fundamental limitations of 2D echocardiographic measurements of VC dimensions, other limitations still remain. In order to measure regurgitant flow rate or flow volume, it would be ultimately desired to measure the different velocities across the VCA accurately in order to determine the products of individual small areas of flow times the individual flow velocity through this area integrated over the entire VCA. Instead of measuring the true spectrum of velocities, current approaches to flow rate only measure the highest velocity across the VCA by means of continuous wave spectral Doppler or calculate the velocity-time integral (VTI) of the highest velocities times VCA to estimate flow volume [29]. As a promising solution of this limitation and a view towards new future technical approaches to measure MR flow, Plicht et al. recently demonstrated that multiple color Doppler aliasing of regurgitant flow at the VCA from a RT3DE color Doppler dataset can be unmasked by dealiasing to accurately calculate absolute regurgitant flow [33]. Alternatively, Skaug et al. described a method based on multiple-beam high pulse repetition frequency (HPRF) color Doppler analysis using 3D color Doppler for accurate automated identification of the VCA and calculation of regurgitant flow [34]. Other limitations that still remain to the representation of the VCA using RT3DE color Doppler belong to a limited temporal and spatial resolution of 3D color Doppler datasets, translation artifacts, and complex dynamic changes of VCA size and shape.

## Basic Principle and Clinical Application of PISA Method Using Color Doppler RT3DE in Mitral Regurgitation

**Fig. 4:**
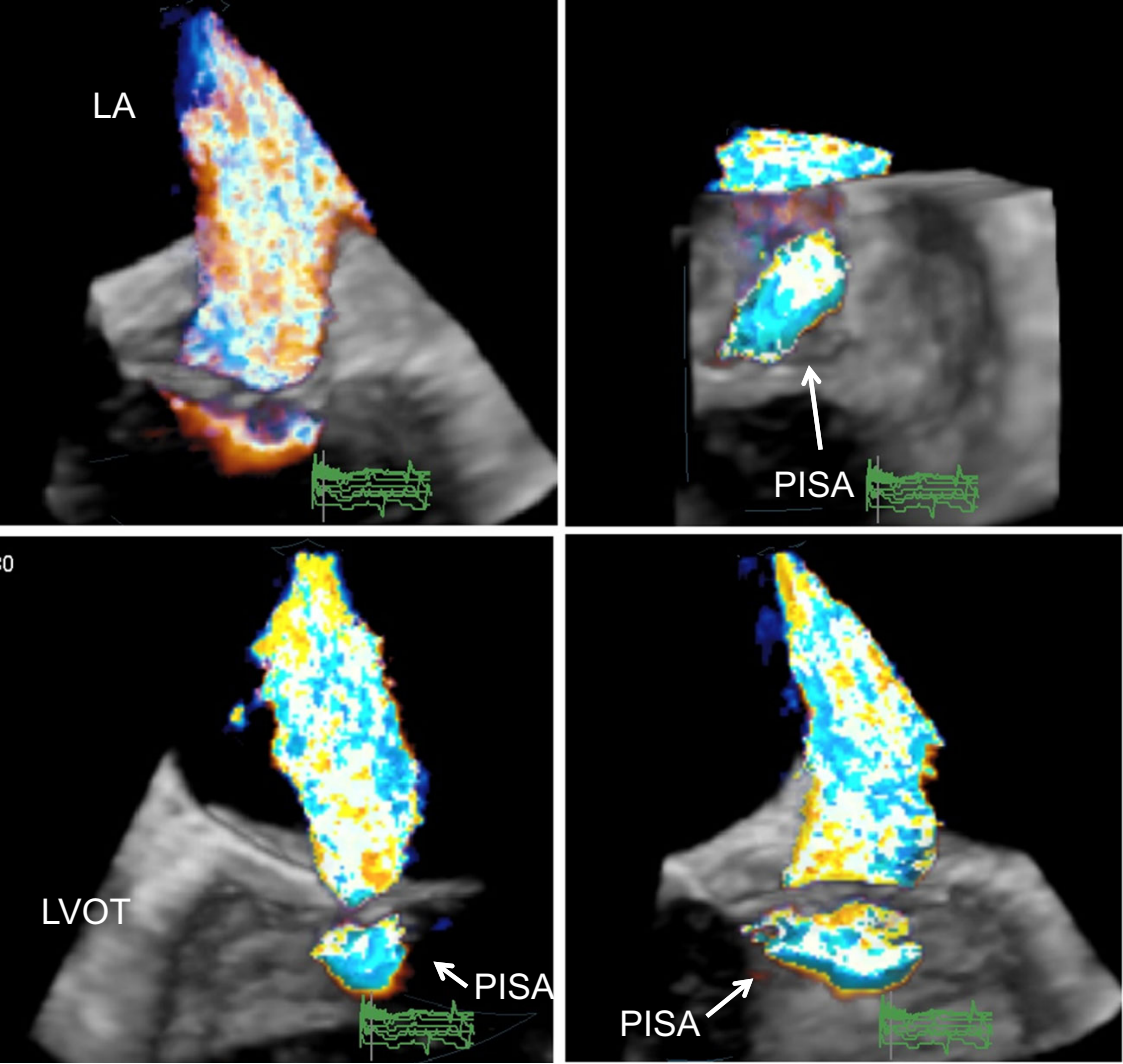
Illustration of the proximal isovelocity surface area (*PISA*) in a RT3D color Doppler TEE dataset of a patient with moderate to severe functional MR. The *figure top left* shows an uncropped view from the LA perspective to the broad jet along the commissure line. *Top right panel* shows a view from the LV perspective to the asymmetric PISA at a Nyquist velocity of 30.8 cm/s. The PISA appears narrow in a long-axis LVOT 3D view (*bottom left*) and broad in a 2-chamber view (*bottom right*). *LA* left atrium, *LVOT* left ventricular outflow tract

**Fig. 5:**
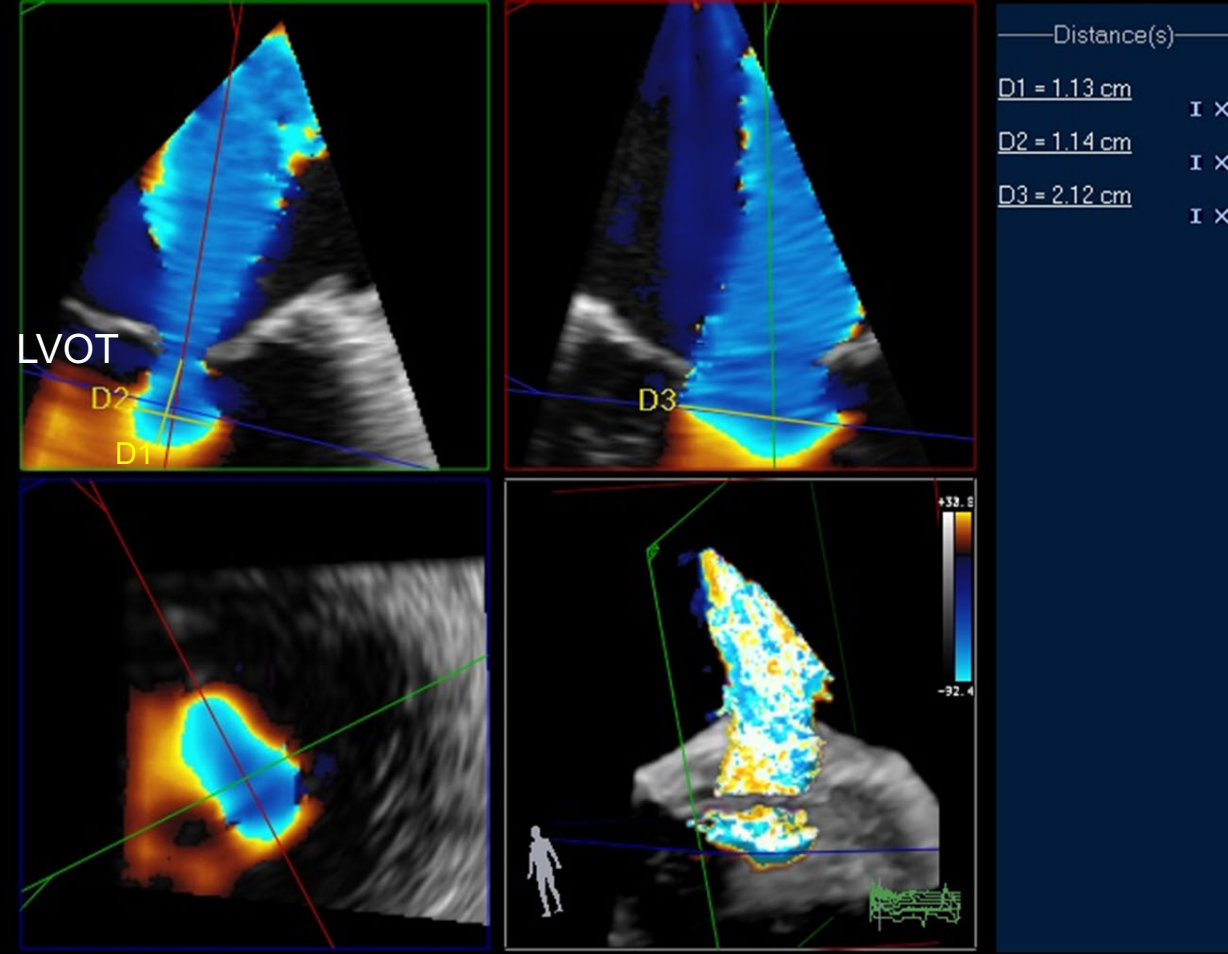
Example of hemielliptic PISA analysis in the same patient presented in Fig. 4 using 3D analysis software (Qlab 9.0, Philips Medical Systems). Hemielliptic PISA calculation is based on the three dimensions of PISA radius indicated as measurement *D1* = 1.13 cm in the *top left panel* (long-axis LVOT view), narrow PISA width (*D2* = 1.14 cm; *top left panel*), and broad PISA length (*D3* = 2.12 cm; 2-chamber view *top right*). PISA calculated using the hemielliptic formula described in the text results at 5.26 cm^2^. EROA calculated with Nyquist velocity of 30.8 cm/s and MR velocity of 420 cm/s results at 0.39 cm^2^. *LVOT* left ventricular outflow tract

Compared to VCA, the proximal isovelocity surface area (PISA) method is hemodynamically more complex. Nonetheless, limitations that pertain to the 2D color Doppler application of the VCA method are similar to the PISA method, which is because of the hemodynamic assumption of a hemispheric shape of isovelocities in the proximal flow field that only holds for a circular regurgitant lesion in unconfined flow [35, 36]. However, as implicated by the asymmetric nature of the VCA, asymmetry should be evident also for the shape of PISA. Early studies using 3D color Doppler datasets already demonstrated that the shape of PISA is not hemispheric but elongated towards a more hemielliptic shape in most cases causing systematic underestimation of EROA and regurgitant flow measured by the hemispheric PISA method [17, 28, 37, 38]. Based on early reports on using a hemielliptic PISA formula [37, 39], more recently, several investigators applied this hemielliptic PISA formula to three orthogonal image planes reformatted from RT3DE datasets and confirmed significant underestimation of MR flow and EROA by 2D hemispheric PISA in patients with non-hemispheric PISA as visualized by RT3DE (Fig. 4) [17, 18••, 33]. Beside the significant underestimation of EROA (*p* < 0.001), Yosefy et al. found clinical important underestimation of the grade of MR severity in 45 % of patients [17]. Kahlert et al. found only small underestimation of 3D-VCA by EROA from 3D-based hemielliptic PISA (mean error −0.09 ± 0.14 cm^2^, *p* <0.001) and described underestimation of EROA by 2D hemispheric PISA to be strongly dependent on the asymmetry of PISA and etiology of MR [18••]. In principle, hemielliptic PISA can be obtained from RT3DE datasets using PISA width, length, and radius for the calculation of the hemielliptic PISA surface by a hemielliptic formula (Fig. 5). The EROA from hemielliptic PISA can then be calculated as EROA = PISA(HE) times Nyquist velocity divided by MR flow velocity as measured by continuous wave Doppler echocardiography. Although the hemielliptic PISA method based on three PISA dimensions is a practical approach to an asymmetric PISA shape, the hemielliptic formula behind is complex and not routinely implemented in current 3D echocardiography systems. A more practical approximation of a hemielliptic surface area is provided by the following formula proposed by Knut Thomsen where *r* is the PISA radius, D1 and D2 are the PISA width and length, and *p* = 1.6075.

$$ \mathrm{HE}-\mathrm{PISA} = 2\pi\ {\left(\left[{r}^{\mathrm{P}}{\left({D}_1/2\right)}^{\mathrm{P}} + {r}^{\mathrm{P}}{\left({D}_2/2\right)}^{\mathrm{P}} + {\left({D}_1/2\right)}^{\mathrm{P}}{\left({D}_2/2\right)}^{\mathrm{P}}\right]/3\right)}^{1/\mathrm{P}} $$

To overcome the limitations of 2D analysis of PISA shape and size, several research groups either validated in vitro or in vivo estimates of the 3D PISA shape by manual measurements of either three perpendicular PISA diameters [17, 18••, 33] or more diameters [40] or PISA surface [41–43, 44•] or investigated computer simulations for semi-automated 3D reconstruction of PISA [45] and found significantly improved accuracy of 3D PISA estimates of EROA and regurgitant flow. An overview of recent clinical studies in which 3D PISA measurements have been compared with other methods of MR quantification is provided in Table 2. Importantly, in a recent study, Ashikhmina et al. demonstrated that direct manual 3D surface reconstruction of asymmetric PISA without geometric assumptions provides significantly larger PISA and EROA (mean 0.44 cm^2^) not only compared to conventional hemispheric PISA (mean EROA 0.19 cm^2^), but also compared to 3D-derived hemielliptic PISA (mean EROA 0.26 cm^2^), suggesting that even the hemielliptic PISA shape is a suboptimal geometric assumption of the asymmetric PISA in functional MR [44•]. All these approaches, however, have not yet found their way into clinical routine application. As a consequence, increasing effort has recently been spent on the development of automated analysis software providing 3D detection of the true PISA surface in the proximal flow convergence zone. Early studies already validated special custom-made computer software for automated detection of the 3D surface of a defined Nyquist velocity in the proximal flow field in 3D echocardiographic patient datasets [37]. Recently, a commercially available method for 3D quantification of PISA without geometric assumptions using single-beat RT3DE color Doppler datasets has been validated in vitro and also described to be feasible in a clinical setting [46•, 47, 48••]. After initial description and validation of this new automated method for quantification of 3D PISA-derived EROA and MR volume [46•, 47], Thavendiranathan et al. recently conducted thorough in vitro validation and clinical validation using RT3D TTE datasets against independent reference methods and found increased accuracy and reproducibility compared to 2DE methods as well as independency from regurgitant orifice geometry [48••]. Using the same automated 3D PISA method, Choi et al. calculated MR volume as EROA times velocity-time integral and found in a differentiated subgroup analysis in 211 patients that MR severity, asymmetrical regurgitant orifice, and eccentric jet were predictors of significant higher accuracy of 3D PISA-derived MR volume compared to 2D PISA using phase-contrast cardiac MRI for reference [49]. The importance of eccentric pattern of the regurgitant jet as a source of significant inaccuracy of 2D PISA measurement compared to 3D-methods was also implicated in the studies by Chandra et al. and de Agustin et al. [47, 50]. However, there is reason for concern that current transthoracic color Doppler RT3DE image quality might not be sufficient enough for valid application of this automated analysis in an acceptable proportion of patients in routine clinical practice. Based on the concept of measuring MR flow volume by integrating the dynamically changing PISA over systolic time as described by our group [51], Thavendiranathan et al. recently applied this automated 3D PISA analysis to a series of color Doppler frames throughout systole [48••]. While presently integration of 3D PISA flow over time has to be performed manually, however, automated integration of 3D PISA flow over systole with appropriately high-color Doppler frame rate would be desired. Using receiver operating characteristic (ROC) curves, Thavendiranathan et al. found a cut-off value of 0.51 cm^2^ for 3D PISA EROA to best differentiate severe from non-severe MR (area under the curve (AUC) 0.91) [48••]. As a notable clinical approach, Schmidt et al. [52•] recently evaluated the same automated 3D PISA method against grading of MR severity based on a weighted integration of routine grading according to current guidelines [6], the MR severity score proposed by our group [21], and the MR index introduced by Thomas et al. [53]. In a receiver operator characteristics curve analysis (ROC) using this integrated metascore, mean EROA determined with automated 3D PISA performed best (AUC = 0.907) compared to peak EROA (AUC 0.840) and EROA calculated from 2D PISA (AUC 0.747). In addition, based on ROC analysis, they found a mean EROA of 0.15 cm^2^ and a cut-off of 0.36 cm^2^ for peak EROA to distinguish severe from non-severe MR.

**Table 2:** Overview of clinical studies validating 3D PISA measurements against 2D methods and independent methods

Study	No. of patients	Scan method	PISA method	Etiology	Comparison method	Correlation/agreement (mean diff. ± SD)	Inter-/intraobserver variability
Yosefy et al. (2007) [17]	50	TTE	HS/HE	Not reported	EROA by 2D QD	HEPISA: *r*² = 0.87 HSPISA: *r*² = 0.59	HE 5.3 %; HS 4.1 %
Kahlert et al. (2008) [18••]	57	TTE	HS/HE	FMR 36 %	EROA by 3D VCA	HEPISA: *r* = 0.96; −0.09 ± 0.14 cm² HSPISA: *r* = 0.93; −0.20 ± 0.20 cm²	–/–
Plicht et al. (2008) [33]	23	TTE/TEE	HS/HE	FMR 47 %	RVol by CMR	HEPISA: *r* = 0.89; −17.4 ± 9.4 ml HSPISA: *r* = 0.81; −11.7 ± 7.4 ml	–/–
Matsumura et al. (2008) [40]	30	TTE	HS/HE	FMR 100 %	EROA by 2D QD	HEPISA: *r* = 0.75; bias −0.10 cm² HSPISA: *r* = 0.69; bias -0.18 cm²	HE 0.06/0.04 cm² HS 0.07/0.03 cm²
Grady et al. (2011) [46•]	33	TTE	Automated 3D PISA	Not reported	EROA by 3D VCA	*r* = 0.61 (*p* = 0.002)	–/–
de Agustin et al. (2012) [47]	33	TTE	Automated 3D PISA	FMR 24 %	EROA by 2D QD EROA by 3D VCA	*r* = 0.96 with 2D QD; −0.05 ± 0.09 cm² *r* = 0.99 with 3D VCA; −0.03 ± 0.04 cm²	ICC 0.96/0.92
Thavendiranthan et al. (2013) [48••]	30	TTE	Automated 3D PISA	FMR 30 %	RVol by CMR	Mean peak PISA: *r* = 0.87; −15.3 ± 12.8 ml integrated PISA: *r* = 0.92; −1.4 ± 9.2 ml	2.2/0.7 ml (integrated PISA)
Choi et al. (2014) [49]	211	TTE	Automated 3D PISA	FMR 47 %	RVol by CMR (*n* = 52)	*r* = 0.97; −0.9 ± 6.9 ml	0.8/0.5 ml
Schmidt et al. (2014) [52•]	93	TTE	Automated 3D PISA	FMR 80 %	Metascore for MR severity	Mean 3D EROA: AUC 0.91 (ROC) Peak 3D EROA: AUC 0.84 (ROC) EROA 2D PISA: AUC 0.75 (ROC)	–/–
Ashikhmina et al. (2015) [44•]	24	TEE	HS/HE/Manual 3D PISA	FMR 100 %	EROA by 3D VCA	Manual 3D PISA: *r* = 0.87; 0.15 ± 0.18 cm² HEPISA: *r* = 0.82; 0.33 ± 0.20 cm² HSPISA: *r* = 0.82; 0.40 ± 0.24 cm²	ICC for all measurements >0.9

*HS* hemispheric, *HE* hemielliptic, *FMR* functional mitral regurgitation, *EROA* effective regurgitant orifice area, *QD* quantitative Doppler, *VCA* vena contracta area, *RVol* regurgitant volume, *CMR* cardiac magnetic resonance, *AUC* area under the curve, *ROC* receiver operator characteristic, *ICC* interclass correlation coefficient

Potential limitations of the existing automated 3D methods being subject of future research include underestimation of convergent flow velocities near the base of the PISA where velocity vectors are almost perpendicular to the vector of the ultrasound beam, dynamic changes of regurgitant flow rate and PISA size during systole combined with dynamic axial and transverse translation of the center of the regurgitant orifice.

## Comparison Between 3D-Derived VCA and EROA by 3D-PISA

There are currently very few studies that directly compared measurements of VCA obtained by RT3DE and measurements of EROA from 3D PISA methods. Ashikhmina et al. found 3D VCA to be statistically significant larger with a mean value of 0.59 ± 0.30 cm^2^ compared to 3D PISA-based EROA with 0.44 ± 0.21 cm^2^ (bias: 0.15 ± 0.18 cm^2^) [44•]. Kahlert et al. did not report absolute mean values but reported 3D VCA to be 0.09 cm^2^ larger than 3D PISA-based EROA [18••]. Using automated 3D PISA detection, de Agustin et al. also reported 3D VCA to be slightly larger by 0.03 cm^2^ compared to EROA by 3D PISA (0.48 ± 0.30 cm^2^ vs. 0.45 ± 0.36 cm^2^) [47]. From these limited data, 3D VCA seems to tend to larger values compared to EROA from 3D PISA, which can be potentially caused by overestimation of the VCA based on the color Doppler representation or by underestimation of the EROA because of underestimation of the true PISA. Cut-off values of 3D VCA proposed to distinguish between severe and non-severe MR are 0.60 cm^2^ reported by Kahlert et al. and 0.41 cm^2^ reported by Zeng et al. [18••, 30]. Proposed cut-off values of 3D PISA-based EROA to distinguish between severe and non-severe MR are 0.51 cm^2^ reported by Thavendiranathan et al. and 0.36 cm^2^ reported by Schmidt et al. [48••, 52•]. The cut-off values from these four studies seem to be significantly different, however, with no clear tendency to larger values by one of the two methods, however, implicating that the two 3D methods potentially might not have the same cut-off values.

## Future Perspectives

In the past years, our knowledge and understanding of echocardiographic flow quantification based on RT3DE color Doppler datasets for estimation of the severity of mitral regurgitation grew continuously. Beside the fact that future methods for flow quantification in mitral regurgitation will likely be based on RT3DE color Doppler datasets, today it cannot be foreseen whether a method based on VCA analysis or a method based on PISA analysis or a totally different method will be validated to be most accurate and feasible. What can be foreseen is that automated software analysis will be developed to allow rapid, robust, and user-independent flow quantification and that such automated flow quantification enabled by increased computer and processing power will encompass the whole dynamic flow information throughout the cardiac cycle. For that reason, also substantial increase of temporal resolution of RT3DE color Doppler datasets is highly demanded. Ideally, such automated analysis software would be capable of auto-detecting the pattern of flow in a defined volume of interest. Thereafter, automated flow quantification should be ideally independent from the shape, size, and number of valve lesions as well as from the spectrum and speed of flow velocities. Several recent semi-automated RT3DE-based methods are promising to reach already closer to this goal. However, it remains unclear whether future automated methods will be based on color Doppler data anyway as they only provide limited spatial and temporal resolution as well as limited accuracy of velocities due to autocorrelation processing. Ultimately, future flow quantification methods might be based on different source data like volumetric spectral Doppler data (as used by the backscattered power-velocity integral method) [54, 55] or vector flow analysis [56] for example.

## Conclusion

RT3DE color Doppler echocardiography has substantially improved our understanding and accuracy of clinical application of the vena contracta area and proximal isovelocity surface area methods in mitral regurgitation. Particularly, the demonstration of the VCA by color Doppler RT3DE to be strongly asymmetric in the majority of patients and etiologies led to a change of paradigm with strong impact on the clinical application of 2D VCA and 2D PISA methods particularly in patients with functional MR and eccentric jets. Because new 3D-based quantification methods effectively overcome the limitations of former 2D methods, definition of new cut-off valves for the estimation of severity of mitral regurgitation particularly based on clinical data is strongly needed. Future advancements to 3D-based flow quantification methods are foreseen to be building on automated and nearly user-independent automated software analysis tools.
